# Tailoring a mobile health text-messaging intervention to promote antiretroviral therapy adherence among African Americans: A qualitative study

**DOI:** 10.1371/journal.pone.0233217

**Published:** 2020-06-09

**Authors:** Elizabeth C. Pasipanodya, Jessica L. Montoya, Caitlin W.-M. Watson, María J. Marquine, Martin Hoenigl, Rogelio Garcia, John Kua, Verna Gant, Joel Trambley, David J. Moore

**Affiliations:** 1 Santa Clara Valley Medical Center, San Jose, California, United States of America; 2 University of California-San Diego, San Diego, California, United States of America; 3 San Diego State University/University of California-San Diego Joint Doctoral Program in Clinical Psychology, San Diego, California, United States of America; 4 Family Health Centers of San Diego, San Diego, California, United States of America; 5 Universal Health Services Southern California Medical Education Consortium, Temecula, California, United States of America; George Washington University, UNITED STATES

## Abstract

African Americans are disproportionately affected by HIV and socio-structural barriers that impact antiretroviral (ART) adherence. Two-way text-messaging interventions have shown promise in supporting adherence in US studies of mostly White people living with HIV (PLWH). However, culturally-appropriate tailoring is necessary to maximize intervention effectiveness among other racial/ethnic groups. Thus, to refine an existing text-messaging intervention, we examined barriers and facilitators to ART adherence among African Americans and perspectives on features to integrate into the extant intervention. Three focus groups, two with African American PLWH (n = 5 and n = 7) and one with providers of care (n = 11) were conducted; transcripts of audio-recordings were thematically analyzed. Adherence supports operated at individual, interpersonal, and structural/environmental levels (e.g., using reminders and pill organizers, wanting to protect partners from HIV, and positive interactions with providers). Adherence barriers also operated at multiple ecological levels (e.g., poor mental health, fear of disclosure of HIV status, and unstable housing). Participant-suggested features for refinement included: i) matching content to participants’ comfort with receiving messages referencing HIV or medication-taking, ii) culturally-tailoring content for African Americans, iii) tracking adherence, and iv) encouraging adherence interactions between patients and providers. Feedback from both patients and providers is foundational to designing effective ART interventions among African American PLWH.

## Introduction

Seventy percent of the 1.2 million people living with HIV (PLWH) in the United States are racial/ethnic minorities and African Americans are most affected by HIV. In 2016, African Americans, representing approximately 12% of the U.S. population, accounted for a substantial proportion of new diagnoses (44%), those living with HIV (42%), and those who died from HIV (52%) [1]. Furthermore, the U.S. HIV epidemic is particularly acute among young gay and bisexual African American men, for whom incidence rates are rising while national rates decline. Indeed, at current rates, 1 in 2 African American men who have sex with men (MSM) will be diagnosed with HIV in their lifetimes (vs. 1 in 7 and 1 in 4 for White and Hispanic MSM, respectively) [2, 3]. African American PLWH also encounter greater challenges along the HIV care continuum, consequently achieving lower rates of adherence to antiretroviral therapy (ART) [4]. As suboptimal ART adherence is associated with adverse consequences (e.g., the emergence of ART-resistant HIV mutations, lower CD4 T-cell counts, and higher rates of AIDS and opportunistic infections), there is a clear imperative to provide support to African American PLWH at risk for nonadherence [5, 6].

Socio-structural barriers, including limited access to health insurance and quality healthcare, poverty, discrimination and stigma, inadequate supportive housing, and insufficient services for mental health and substance use concerns, have been identified as important factors reducing rates of ART adherence among African Americans [7, 8]. Although providing in-person support (e.g., through adherence education and counseling, case management and service linkage, and evidence-based therapy) improves ART adherence among PLWH, the infrastructure to provide augmented support is often limited [9–14]. Furthermore, many of the previously-stated socio-structural barriers that impact adherence also limit utilization and reach of auxiliary healthcare services, necessitating alternative methods of providing support [15, 16].

With the ubiquity of mobile phones, mobile Health (mHealth) technologies are alternatives with the potential to promote the health of a broad-range of PLWH. Indeed, cellphone ownership is high and near-universal among African Americans, with approximately 98% of adults possessing at least a basic phone and 75% owning smartphones [17]. Furthermore, prior research has shown text-messaging interventions to be efficacious, low-cost, and scalable in supporting ART adherence [18–22]. The individualized Texting for Adherence Building (iTAB) intervention is an example of a previously-developed, personalized, bidirectional, automated text-messaging system addressing barriers to ART adherence. Recent studies have demonstrated the feasibility and efficacy of iTAB among PLWH, including those with co-occurring methamphetamine use disorder, co-occurring bipolar disorder, and among those at risk for HIV infection [23–25].

Much of the evidence supporting two-way text-messaging for ART adherence among US populations was accumulated in studies of predominantly White participants; however, interventions should be appropriately-tailored to meet the needs of specific PLWH populations to enhance their impact [22, 26]. Multiple tailoring approaches have been described, including matching materials to align with a population’s “surface” characteristics (e.g., race, language, gender, and education); however, fit is likely best improved by also considering target health behaviors in the context of socio-cultural values using input from community members themselves [26, 27]. Indeed, literature reviews of text-messaging interventions suggest larger effect sizes when participants contribute to generating personalized content that incorporates their preferences [22]. Thus, prior to implementing the iTAB intervention among African American PLWH, it was necessary to obtain input from representative individuals and identify areas relevant for tailoring.

The present study used focus groups to understand barriers and supports to ART adherence from the perspectives of African American PLWH and their providers of care. Additionally, we solicited and obtained feedback to modify an extant intervention (iTAB) to match the expressed needs and concerns of African American PLWH.

## Materials and methods

### Participants and procedure

Participants were patients and providers from Family Health Centers of San Diego (FHCSD), a large community health organization in Southern California, recruited to participate in the formative phase of a longitudinal text-messaging study to support ART adherence among African American PLWH. FHCSD is a private, 501(c)(3) non-profit community health center whose mission is “to provide caring, affordable, high quality healthcare and supportive services to everyone, with a special commitment to low income” [28]. Indeed, more than 96% of patients seen at FHCSD are at or below 200% of the Federal Poverty Guidelines [29]. With nearly 40 clinics and programs and a staff of well over 1,000, FHCSD provides care annually to more than 210,000 unique patients. FHCSD offers a wide range of health care services, including HIV care, primary care, dental and vision, behavioral health, outpatient substance abuse, mobile medical, and pharmacy services. FHCSD is the largest provider of comprehensive HIV/AIDS services and of healthcare to uninsured patients in the San Diego region and, as a Ryan White Part C-funded provider of HIV medical care and support services, FHCSD provides HIV care to over 1,200 patients living with HIV per year [28, 29]. The patient population at FHCSD is represented by a wide range of ethnic/racial groups, including 52.4% Hispanic, 25.4% White, 11.3% Black/African American, 4.7% Asian, 1.5% American Indian/Alaskan Native, 1.1% Native Hawaiian, and 3.7% more than one race. Only 77% of African American PLWH receiving care at FHCSD are virologically suppressed, compared to 90% across the entire non-AA clinic population (with similar rates of virologic suppression among non-Hispanic Whites and Hispanics).

Patients and providers participating in this study were recruited from two FHCSD clinics located in close proximity to each other that have an emphasis on providing services to PLWH. The study protocol was approved by the institutional review board (IRB) at the University of California, San Diego (IRB project number– 161608) and participants provided written informed consent.

Potential patient participants were identified by staff at FHCSD through reviews of scheduled appointments and reviews of the electronic medical records of individuals potentially-meeting study criteria. Identified individuals were approached at their visits or telephoned and invited to participate. Patient inclusion criteria were i) African American/African ancestry, ii) English-speaking, iii) age 18 or older, iv) diagnosis of HIV and ART prescription, and v) ability to provide informed consent. Provider participants were invited to participate following announcements in staff meetings. Provider eligibility was the provision of services (medical or psychosocial) to African American PLWH on ART.

Twelve African American PLWH participated in two patient focus groups (n = 5 and n = 7, respectively) and 11 providers of clinical care participated in a provider group between February and March 2017. Empirical research on the number of focus groups to use in qualitative studies with nonprobability suggests that more than 80% of themes are discoverable within two to three focus groups, so the total number of focus groups in this study was deemed adequate to achieve sufficient coverage of themes [30]. The median (IQR) age of patients was 47 (34, 51) years and most were cisgender male (83.3%). Regarding providers, the median (IQR) age was 44 (34, 47) years old and most were female (54.5%). Providers were racially/ethnically-diverse, with 36.4% identifying as White and the rest African American, Asian, or Mixed Race; 45.5% were Hispanic/Latino. Lastly, a substantial proportion identified their service areas as primary care, with either RN or MD qualifications (27.3% each), and the remainder social work/case management.

### Focus groups and analyses

Patient and provider groups lasted 60 to 90 minutes and were held in a conference room at FHCSD. Light meals and refreshments were provided for all participants; patients were compensated $20 in gift cards. Discussions were led by study team members with expertise in facilitating focus groups.

Using a semi-structured guide, each focus group explored supports and barriers to ART adherence encountered by African American PLWH and suggestions to tailor the intervention. For instance, in discussions of barriers to adherence, patient participants were asked, *“In your experience*, *what has gotten in the way of taking your anti-HIV medications*?*”* For views on text-messages, they were asked, *“What kind of messages do you think would encourage taking medications*?*”* Providers were posed corresponding questions.

Focus groups were audio-recorded and transcribed verbatim, without identifiable information. Subsequent to transcription, a thematic analysis approach was carried out [31, 32]. Transcripts were independently coded by two investigators using MAXQDA and a coding dictionary of mutually-exclusive code definitions and memos was constructed [33]. Initial interrater reliability was low, resulting in further iterations of code refinement and assignment. Disagreements were resolved through establishment of consensus and, following additional review, final inter-rater reliability was high (Cohen’s kappa > 0.9) [34].

## Results

### Facilitators to adherence

Table 1 presents themes and exemplar quotes emerging from discussions of facilitators to adherence. Three thematic facilitators, operating at differing ecological levels of influence to impact adherence, were identified: (i) individual facilitators, (ii) interpersonal facilitators, and (iii) structural/environmental facilitators.

**Table 1:** Patient perspectives on supports to adherence.

Themes	Exemplar Quotations
**Individual Facilitators**	
**Cognitive**	
• To maintain health and appearance / take care of self • To live • To avoid drug resistance or deterioration of health • Belief in medication efficacy	• *“…you know*, *I’d rather keep what I’ve got than trying to get back what I’ve lost ‘cause I’ve seen people who*, *I guess*, *waited too long*, *and they just*, *I mean*, *physically just aren’t the same and that just*, *and*, *you know*, *I’m vain and I’m just*, *I’m real vain and as far as I only get one meat suit*, *I want to try to keep this intact as long as I can*.*”* (Patient Participant) • *“… it’s more of a cognitive reinforcement thing to get the undetectable result back*, *it’s like*, *‘Oh*, *you’re doing a really good job*.*’”* (Provider Participant)
**Behavioral**	
• Using memory aids or reminders • Using pill organizers • Establishing routines • Carrying extra pills and planning ahead	• *“I have to do it every morning*, *have breakfast and take my medication*. *When I eat*, *I take it then and I do it every morning at breakfast time*. *I will not miss*, *whatever I do*, *I will not miss breakfast*, *‘cause breakfast is the only meal I will eat (laughter) throughout the day*. *But breakfast and my medication goes hand-in-hand and that’s what I do*.*”* (Patient Participant) • *“Some patients have pillboxes that have multiple doses in the day*.*”* (Provider Participant)
**Interpersonal Facilitators**	
• To protect partners • Social support	• *“I want to have sex*. *I want to date*. *I’m*, *you know*, *I’m not dead yet*, *you know what I mean*? *So*, *I want to have*, *I got to do what I can to protect those I’m gonna be with ‘cause I was like*, *‘I don’t want my love to kill you*,*’ you know*?*”* (Patient Participant)
**Patient-provider interactions**	
• Provision of informational support • Frequent patient monitoring (e.g., for bloodwork) • Open communication and problem-solving • Processing patient experiences and reflecting back their values • Team approach to medical care and development of relationships with patient	• *“… it was just really detail oriented*. *Like no question left unanswered*. *And if he did*, *and if there was a question that he had to get answered*, *you know*, *he would*, *it was cool*, *and the information was right there for you*.*”* (Patient Participant) • *“So many patients often say*, *‘Oh*, *I don’t know my CD4 count is*,*’ or*, *‘Nobody explained that to me*.*’ So*, *I think taking the time to engage the patient and their healthcare and knowing and understanding what their blood work actually means*.*”* (Provider Participant)
**Structural/Environmental Facilitators**	
**Medication/treatment-specific**	
• Intensive medication management • Simplified regimen	• *“The reason I started taking pills is because I was*, *I was in a treatment facility and from the treatment facility*, *you know*, *I came back from the facility with the medication on my person*.*”* (Patient Participant) • *“And then the simplicity of just*, *you know*, *taking one pill a day*, *I think that helps a lot with our clients too*.*”* (Provider Participant)

#### Individual facilitators

Individual-level facilitators fell into two broad categories of cognitive and behavioral facilitators. Cognitive facilitators were intrapersonal motivational reasons for ART adherence. For instance, most patient participants spoke of the desire to maintain good health and stave off negative changes to physical appearance. Providers similarly cited maintaining gains achieved by consistent adherence as a motivator for adherence among their patients.

Participants additionally identified behavioral facilitators, corresponding to intentional actions to enhance memory and increase the probability of adherence. Providers and patients both highlighted the utility of memory aids (e.g., phone reminders or alarms) and pill organizers, with most patients indicating having used memory aids in the past. Patients uniquely reported establishing daily routines, pairing medication-taking with behaviors, and carrying pills.

#### Interpersonal facilitators

A subset of themes revolved around relational supports to adherence. For instance, some patient participants reported taking ART to protect their sexual partners from infection, stating *“I don’t want my love to kill”*. Several patients additionally reported receiving social support from family, who remind them to take ART. A set of themes that were widely endorsed by patient participants were related to supportive patient-provider interactions. Participants identified informational support from providers as helpful for understanding HIV and supporting ART adherence. Many providers additionally reported facilitating open communication and endorsed engaging in active problem-solving as helpful for bolstering adherence.

#### Structural/Environmental facilitators

Operating outside the individual and their relationships with close others were contextual factors. A limited set of medication/treatment specific themes were raised. A few patients reported it helpful to initiate and sustain medication-taking in intensively-managed treatment settings (e.g., within substance abuse programs) while many providers described a one-dose-a-day regimen as facilitating adherence.

#### Barriers to adherence

Table 2 presents themes and representative participant quotes reflecting barriers to ART adherence. As with facilitators, themes related to barriers were: (1) individual, (2) interpersonal, and (3) structural/environmental.

**Table 2:** Patient and provider perspectives on barriers to ART adherence.

Themes	Exemplar Quotations
**Individual Barriers**	
**Poor mental health**	
• Substance Use • Depression • Feeling overwhelmed	• *“So*, *I’m a recovering addict*. *And when I was on the good one… those pills are gonna have to wait*. *If that means I miss my doctor’s appointment*, *I miss all these other things and… that took up most of my life*.*”* (Patient Participant) • *“Mental health and substance abuse*. *Those things play a really big role in adherence*.*”* (Provider Participant)
**Behavioral Barriers**	
• Difficulty establishing a routine • Running out of refills	• *“I don’t know when the best time to take it is*, *so that is like one problem I have… and I’m a free spirit*. *Like*, *you know*, *I’m not structured like that*. *I’m not wired that way*. *Although*, *like I’ll do what I need to do but I’m free flowing*, *you know what I mean*?” (Patient Participant) • *“One place we seem to lose some people is getting refills*, *especially in the last refill*, *that they didn’t know they had another one… It’s just one*, *some of the people who come in with a viral load are people who were undetectable*, *didn’t get their last refill*, *couldn’t/wouldn’t get an appointment*, *come in and try to see somebody else*, *and we lose two or three weeks of medicines there*.*”* (Provider Participant)
**Cognitive Barriers**	
• Forgetting • Dislike of medications • Burden of life-long therapy • Denial	• *“As for me*, *after the fifth year*, *it became very hard because I realized you’re takin’ this for a whole lifetime and it gets very frustrating*. *It gets like you just want to forget the whole thing because you’ll never be normal again*. *And that sticks in your mind sometimes because everybody else not doing it but you’re doing it*.*”* (Patient Participant) • *“… taking one pill a day is easy to forget or*, *‘Did I just take that or not*?*’ because it becomes so second nature to you*.*”* (Provider Participant)
**Medication and Symptom-Related**	
• Side effects • Pill burden • Feeling well	• *“… the first six years*, *I mean*, it was kicking my a** if I didn’t eat, *you know*? *I mean*, kicking my a** and I’m going, *‘This is to stay alive*? *Every day*?*’”* (Patient Participant) • *And most of these people are feeling just fine so there’s not a lot of negative reinforcement like*, *‘I missed a dose this week*. *I feel fine*. *Maybe I could miss two next weeks*.*’”* (Provider Participant)
**Interpersonal Barriers**	
• Poor support from others • Stigma and fear of disclosure	• *“But I tell my best friend*, *just the other day I was telling her like*, *‘You know*, *no one ever talks about this*. *Like and you guys don’t*, *so and it would mean a lot to me if you were to just acknowledge it every once in a while because you acknowledging it*, *makes it real for me*.*’ Because I could be in my own fantasy world where I don’t have it because I’m so*, *I’m*, *you know*, *I’m just living life like everybody else*, *just doing my thing*.*”* (Patient Participant) • *“I know some people just can’t have that discussion around HIV or even medical care*. *And I know that in some cultures… I mean*, *just if it’s a female HIV-positive*, *African-origin woman*, *she can’t have that discussion with anyone outside*.*”* (Provider Participant)
**Structural/Environmental Barriers**	
• Homelessness/Unstable housing • Problems with insurance • Problems accessing health care	• *“I’m in a homeless program right now… And we have to be off the floor at 9*. *So*, *there’s a lot of rushing around*. *And even though I have my pills in the little thingy*, *I sometimes don’t even have time to pop that little thingy out because I’m worried about if I have all my right paperwork*, *if I have everything that I need*, *because I’m not gonna be able to come back on this floor until so and so time… It’s always the first thing on my mind*, *but it always gets put on the back burner when I’ve got to make sure I’ve got everything that I need*, *because I get downstairs and think*, *‘What about my meds*?*’”* (Patient Participant) • *“A lot of our homeless clients have to worry about their medication being stolen or lost*.*”* (Provider Participant)

#### Individual barriers

Participants identified individual-level barriers falling into four categories: cognitive barriers, behavioral barriers, poor mental health, and medication and symptom-related factors. A common cognitive barrier, listed by both patients and providers, for inconsistent adherence was forgetting. Some patients also spoke of adherence as affected by a dislike of medications (i.e., that they were not *“pill [people]”*) and by the burden of lifelong ART. Patients also described ART as a reminder that they would “*never be normal again*” and reported nonadherence as maintaining avoidance of thinking about living with HIV.

With regards to behavioral barriers, both providers and patients identified missing appointments and subsequently running out of refills as a barrier. Additionally, several patients reported difficulties with establishing routines, with some describing themselves as *“free spirits”* reluctant to maintain consistent dosing times. Others reported difficulty integrating regimented dosing with busy work and family schedules.

Patients additionally reported medication and symptom-related barriers to adherence. For instance, most patients reported experiencing side-effects and needing to take medications with food as contributing to nonadherence. Additionally, a few individuals reported pill burden, particularly when ART was combined with other prescriptions. Both patients and providers identified good physical health as sometimes contributing to nonadherence because of perceived leeway to miss doses without consequence.

#### Interpersonal barriers

Despite reporting social support as helpful, participants also described relational and social barriers. In particular, most patient participants described nonadherence as sometimes resulting from efforts to maintain confidentiality about HIV status and avoid stigmatization–“*Most of us*, *well*, *I would say most of us ‘cause*, *comin’ from the south*, *we don’t want the other person to know we have HIV*, *so we refuse to take it…*”.

Some patient participants additionally reported receiving unsupportive responses from trusted individuals and avoiding soliciting support from those close to them because of reticence among African Americans to discuss health and medication-taking–*“Now you can talk about anything else*, *ANYTHING ELSE… Okay*, *I know not to say anything around this buddy about meds or stuff like that*, *you know*?*”*

#### Structural/Environmental barriers

Patients and providers also acknowledged several structural and environmental barriers to adherence. Many participants described the instability inherent to homelessness as contributing to inconsistent adherence and to lost or stolen medications. Furthermore, a few participants described difficulty accessing healthcare, primarily due to insurance problems.

### Tailoring the intervention

#### Suggestions for the intervention

Table 3 presents representative quotes and themes relevant to tailoring iTAB. Both patient and provider participants reported concern that receiving adherence messages carried risks of unwanted disclosure of HIV status and of medication-taking. Thus, they all unanimously emphasized discreet adherence messages to mitigate risks. Providers additionally suggested matching the degree to which messages referred to HIV or medication-taking to participants’ levels of comfort with receiving such messages.

**Table 3:** Participant feedback relevant to tailoring the intervention.

Themes	Exemplar Quotations
Limiting the unwanted disclosure of HIV status or of medication-taking	• *“I would probably have something that no one would see… It has to be something that–I don’t want anyone else to know but me*.*”* (Patient Participant) • “*So*, *I mean*, *it sounds to me like you can kind of gauge the zero disclosure to high disclosure type person*, *and then*, *you know… go from there*.*”* (Provider Participant)
Personalized messages (choice in timing and content; ability to select from set domains or write one’s own messages)	• *“When you set your reminder for through whichever system you want to use*, *you know*, *there should be an option for you to be able to personalize and customize your message that you’re gonna receive*, *you know*, *‘cause it’s your own personal message*.*”* (Patient Participant) • “*There might be room for premade bundles to choose from*, *sort of*. *In the sense like*, *I know people who*, *who if they got a sports score on their phone*, *they have a feed all the time*, *nobody would blink*, *nobody would know it meant anything but*, *you know*, *if all it has to do is remind them*, *then knowing that this came from a specific thing and it comes every day would work*. (Provider Participant)
Providing culturally-relevant content	• *“… facts not related to HIV that apply to the African American community*.*”* (Provider Participant)
Ability to “snooze” messages and receive additional reminders	• *“Whereas something that would say*, *‘Have you taken your med yet”” And then they have*, *they’re a little bit more engaged—say*, *‘Yes*, *no*, *or I will*. *Remind me in five minutes or something*.*’”* (Provider Participant)
Ability to track and monitor adherence over time	• *“Well*, *it may be a way of tracking it*, *too*, *‘cause they may tell you they missed twice and it might be six times in the month but they remember twice… So maybe for them to see in the last month*, *be able to like go back and see*, *‘Oh my gosh*, *like I actually see it now*, *like this many times*.*’ And so it may bring a little bit of accountability for them*.*”* (Provider Participant)
Receiving reinforcement for adherence	• *“Maybe something–I’m thinking of a concept of like kindergarten gold star thing*. *Like you do something good*, *and you get gold stars*.*”* (Patient Participant)
Facilitating interactions with medical providers centered around adherence	• *“Text messages can lead into that or it could be a part of*, *a branch of that*, *you know*? *But the face-to-face and the doctor aspect of it also needs to be implemented into that for like the full picture*.*”* (Provider Participant)

Regarding the content of messages, patient and provider participants largely all agreed and suggested that adherence messages vary and that they be positive/inspirational, educational, entertaining, and relevant to health. Several participants also discussed personalizing messages, with customizable messaging (i.e., with choice in message type and the ability to compose messages) and timing (such that messages could be received at participant-selected times). Furthermore, a few participants suggested culturally-tailoring messages with content specific to African Americans.

A number of participants additionally suggested a feature allowing tracking of adherence over time and that the intervention promote adherence discussions between patients and their medical providers. Other features many participants recommended included “snoozing” messages to allow additional reminders and reinforcement messages acknowledging positive adherence responses.

#### Resultant changes to the text-messaging intervention

Table 4 presents message suggestions, extrapolated text-message domains, and sample text-messages incorporated into iTAB. Domain areas, emanating from focus groups and adapted from previous iTAB iterations, included 1) celebrate health, 2) dangers of non-adherence, 3) dose-time focus, 4) social support, 5) self-esteem and positive affirmations, 6) religious/spiritual messaging, 7) health facts, and 8) trivia. Messages celebrating health were approach-motivated, highlighting positives of adherence to health while messages in “dangers of non-adherence” highlighted potential negative impact of nonadherence. Dose-time focus messages were direct reminders alerting individuals to take ART while social support messages highlighted social reasons to motivate adherence. Self-esteem messages promoted self-worth while religious/spiritual messages were encouragements of adherence drawn from multiple faith communities. Lastly, health facts and trivia were domains providing health-related information and general facts, respectively.

**Table 4:** Participant suggestions, extrapolated domains, and sample text messages incorporated into the iTAB intervention.

Participant Message Suggestions	Extrapolated Domains	Representative intervention text messages
*“Don’t forget to take care of yourself today*.*”* (Provider Participant)	Celebrate Health	“To help keep you feeling good, this is a friendly reminder to be mindful of your health today.”
*“Something like bubbles or dots*. *And then a bubble up saying like*, *‘The time is now*.*’”* (Patient Participant)	Medication Time Focus	“It’s show time!”
*“… you are not alone*! *Remember to identify and reach out to the support networks that are all around you*.*”* (Provider Participant)	Social Support	“You are an important person to the people around you!”
*“I think maybe some*, *I don’t know how you could teach or bring about optimism and hope and*, *you know what I mean*?*… some positivity*.*”* (Patient Participant)	Self-esteem/Believing in yourself	“Each day, you are one more step in the right direction.”
*“You could send bible verses*.*”* (Provider Participant)	Religious/Spiritual	" “We need God as much in the calm as in the storm.” "–Jack Hyles.
*“How about RIP*?.* *.* *. *Maybe like put some fear in you*.*”* (Patient Participant)	Dangers of Non-adherence	“You need to be healthy to live long.”
*“Maybe a word of the day*.*”* (Patient Participant)	Trivia	“laconic: Expressing much in few words; concise”

The iTAB intervention was also tailored to allow future participants choice in receiving either “high” or “low” disclosure messages, with “high” disclosure messages containing references to either HIV or medication-taking and “low” disclosure messages containing references only to health and well-being (i.e., no mention of HIV/medications). An example “high” disclosure message is "Your health matters! It’s dose O’clock", whereas a corresponding “low” disclosure message simply states, “Your health matters!” In addition to selection from multiple domains within these levels of comfort, the iTAB system was tailored to allow future participants the ability to compose their own messages. Furthermore, messages were also tailored to include culturally-relevant messages, with historical and contemporary facts pertinent to African Americans.

**Fig 1:**
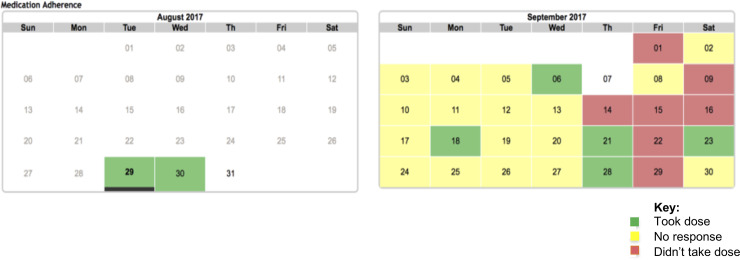
Sample adherence calendar for a test participant receiving one iTAB adherence message daily. Adherence calendars are based on self-reported adherence via the iTAB text-messaging system. Test participant received adherence text messages between 08/29/17 and 09/30/17; no message was sent out on 09/07/17.

To facilitate tracking adherence over time, the iTAB system was scripted to generate an adherence “calendar”, capturing adherence responses received, that could be presented to participants at in-person visits (Fig 1). Incorporating the suggestion to facilitate interactions between patients and their providers, participants in future iTAB studies would also be provided with the choice of sharing these calendars with their providers to promote conversations around adherence.

No changes were made with regards to suggestions for features already in the existing intervention (i.e., choice in message content and timing, varying messages, a “snooze” feature, and reinforcement messages).

## Discussion

This study endeavored to fill a gap in the literature by using qualitative methods to identify barriers and facilitators to ART adherence among African Americans and to obtain key stakeholder perspectives on appropriately-tailoring an ART adherence intervention. Thematic analyses of focus groups involving African American PLWH and providers of care indicated that barriers and supports to ART adherence operate at multiple ecological levels (i.e., individual, interpersonal, and structural/environmental) to impact ART adherence. For instance, participants discussed the positive influence of using memory aids and pill organizers, the desire to protect sexual partners, and receiving support from providers. They also noted the negative impact of factors such as poor mental health, erratic routines, and unstable housing. Overall, many of the barriers and supports to ART adherence discussed in the focus groups are in line with those that have been described in other settings and populations that experience adherence difficulties [35]. For instance, recent work among PLWH in the US has similarly identified the negative influence of factors that include stigma, mental health concerns, unstable housing, poor healthcare access, and competing life activities, as well as the positive influence of other factors like positive health beliefs, social support, and the use of memory aids [35–38].

Although emerging themes were similar across patient and provider focus groups, the density, depth of description, and variety of subthemes at each of the ecological levels occasionally differed among patients and providers. For instance, patient participants discussed considerably more individual-level barriers and facilitators than providers did, while providers cited more interpersonal and environmental facilitators and barriers related to the experience of accessing healthcare. Additionally, despite better tolerability and lower side effect profiles of contemporary ART, patients uniquely reported the continued experience of side effects and the need to take some formulations of ART with food as barriers to adherence. Furthermore, salient among patients but not among providers was an awareness of ambivalence, or dialectical tension experienced by individuals taking ART. Specifically, both patients and providers cited the desire to maintain physical health as a facilitator of adherence; however, only patients noted that taking ART was a daily (unwelcomed) reminder of HIV, with nonadherence sometimes functioning as a form of avoidance of thinking about living with HIV. Thus, the degree of elaboration around barriers and facilitators across groups tended to follow how proximal and familiar experiences were to discussants.

Feedback from patients and providers suggested innovations to improve the iTAB intervention and its fit with African American patients’ needs and preferences. For instance, across both groups, participants suggested having choice in the degree to which text-messages discussed the presence of HIV or a health condition, as they noted varying comfort levels with potential disclosure of health information. Such privacy concerns echo those previously reported in qualitative work among African American PLWH during the development of technology-based interventions for promoting retention in care [39, 40]. Specifically, African American PLWH have stressed the importance of protecting sensitive personal information, such as sexuality and HIV serostatus, and allowing for anonymous participation to mitigate risks of inadvertent disclosure [39, 40].

Participants also suggested tailoring the iTAB intervention by incorporating trivia messages with content directly referencing or related to African Americans. Additionally, to facilitate monitoring ART adherence, focus group discussants suggested including a feature to track ART adherence over time. This was accomplished by scripting the iTAB system to generate adherence “calendars” that will be presented to future participants at in-person visits. Relatedly, providing feedback on ART adherence has been found efficacious in improving adherence across several studies that collected adherence data using electronic drug monitors [41–43]. Indeed, the evidence to support using feedback as an adherence tool was found to be compelling enough for recommendation of feedback via electronic drug monitoring as an evidence-based intervention for supporting ART adherence [44]. Although our calendars will relay summaries of self-reported adherence, there is potential that this patient-driven feature may support future adherence. Lastly, incorporating feedback to promote conversations between patients and their providers of care, participants in future studies will also be provided with the choice of sharing these adherence calendars with their providers. Altogether, building upon prior work [45, 46], we refined the iTAB intervention for application among African American PLWH by soliciting for and acknowledging the needs and concerns of African American PLWH and their providers of care. Interpretation of our study findings, however, should consider its limitations. First, tailoring the text-messaging intervention was circumscribed to influencing individual and interpersonal barriers of adherence; thus, the iTAB intervention may not address barriers to adherence that operate at other ecological levels. Second, the small participant sample size and relatively small number of groups limit generalizability of findings and may preclude saturation, particularly with respect to themes relating to the differences between patient and provider perspectives. Additionally, our sample was predominantly cisgender male; thus, we were unable to explore how gender may act to impact adherence to ART among African Americans. Furthermore, by only using focus groups, our work may not reflect the same degree of insight as it might have using a combination of in-depth key informant interviews and focus groups. Future work should consider utilizing a larger, more gender-diverse sample as well as a multimethod qualitative approach. Furthermore, discussions relied on retrospective reports, which may not be an accurate measure of factors associated with adherence behaviors. Lastly, the amenable response to using technology to promote adherence may be an artifact of selection bias introduced during convenience sampling.

In summary, our findings offer significant contributions to the literature by exploring both patient and provider perspectives of barriers and supports to ART adherence among African Americans. Additionally, consistent with participatory-based design, we solicited input relevant to tailoring the iTAB intervention from key stakeholders and conducted formative research involving the community with which future work will be conducted [47]. Future research will investigate whether this translation into a personalized and tailored intervention results in improved ART adherence behaviors among African American PLWH.

## Supporting information

S1 Text Patient focus group guide. (DOCX)
