# Supplementary material for: Tailoring a mobile health text-messaging intervention to promote antiretroviral therapy adherence among African Americans: A qualitative study
Source: PLoS One. 2020 Jun 9;15(6):e0233217. doi: 10.1371/journal.pone.0233217 (PMC7282643; doi:10.1371/journal.pone.0233217)
Supplement: S1 Text — (DOCX) [file pone.0233217.s001.docx]

**Patient Focus Group Guide**

**Adherence**

Most people living with HIV take anti-HIV medications and, we would like to figure out ways to maximize how well people take their medications. Now, thinking about the medications that you may take…

1. What do you do to help yourself to remember to take your anti-HIV medications?
2. Are there other things about you (like personal beliefs or feelings) or in your environment that help you take your medications? How has this changed for you since you were first diagnosed?
3. What do you like about these methods; what make them ideal for you?
4. In addition to your personal experience, have you observed or do you know of, other factors that support taking anti-HIV medication? If so, what are these things?
5. What do you think might be particularly helpful for members of the AA community, relative to other groups, when it comes to taking anti-HIV medications as prescribed?
6. What sorts of things get in the way of taking your anti-HIV medications as prescribed? (probes: mood, drugs/alcohol, pain, fatigue, childcare, etc.)
7. Even if it hasn’t been your personal experience, have you seen or do you know of other factors that get in the way of consistently taking anti-HIV medication? If so, what are these things?
8. Are there things that you think might be a big problem for AAs, relative to those in other communities?

**Mobile/smart phone usage**

As previously mentioned, in our proposed intervention, part of the plan will involve sending text messages to cell phones to help people remember to take their anti-HIV medications.

1. We’d like to ask you some questions about cell phones.
   1. How many of you own a cell phone? A smartphone?
   2. Do you keep it on and carry it with you daily?
   3. Do you share your phone with any other member of your family or with friends?
   4. How often do you use your phone to text? For what purposes do you use text messages?
   5. Do you or your friends/family always have minutes? Do you or your friends/family have prepaid plans?
   6. If you don’t have a phone, why not? Did you ever have one?
2. In our study we will ask people to carry a mobile phone everyday for 48 weeks that would text them with reminders to take anti-HIV medications and they will respond to those messages. The idea is that intensive texting will serve as the main way to help people adhere to anti-HIV medications.

Would you, or other people that you know, be willing to participate in this kind of study?

1. Why, or why not, would you consider participating in a study like this one? What might help motivate other people to participate in this study?
2. How would you feel about receiving text messages every day to remind you to take your medication?
3. How can we make it as enjoyable as possible to receive daily texts?
4. Do you think your partner or other family members would be ok with you receiving daily texts for the study?

**Text Message Content**

Now we would like to shift gears a little to open up a discussion of what the text messages should say. We would really want this text messaging system to serve as the main support for people to be adherent to their anti-HIV medications. We have other things like counseling to help, but we really would like to understand how to tailor the messages to help keep people on track.

1. What kind of messages do you think would encourage people like you to take their medications?
2. Would you be interested in specific types of messages regarding taking your medications? (probes: positive/upbeat, short/ long, health-focused, overcoming barriers, drawing on support, etc)…or more interested in general reminders? Are there any types of messages you would not like to receive?
3. Over the next minute or two, please write down about 5 messages that you think would be useful for people to remember to take their anti-HIV medications

**Conclusions**

1. Does anyone have anything else they would like to add to our discussion group today?

2. Are there any other things that you think would be helpful for anti-HIV medications adherence?

Thank you very much for your time today and for your feedback.

**Provider Focus Group Guide**

**Adherence**

1. From your perspective as providers, what factors get in the way of adherence to ART? (probes: mood, drugs/alcohol, pain, fatigue, childcare, etc.)
2. Does the AA community you serve have any unique factors that impact their adherence to ART, either positively or negatively?
3. Are there specific things that you do or suggest to to help your patients remember to take their anti-HIV medications (calendars, pill boxes, reminders, texts, etc)?
4. What do you like about these methods; what make them ideal for your patients?

**D. Recruitment and mobile phone usage**

As previously mentioned, in our proposed intervention, part of the plan will involve sending text messages to cell phones to help people remember to take anti-HIV medications.

In our study we will ask people to carry a mobile phone everyday for 48 weeks so that we can text them with daily reminders to take anti-HIV medications and also receive daily responses about medication adherence in return. The idea is that intensive texting will serve as the main way to help people adhere to their anti-HIV medications.

1. Do you think that your patients be willing to participate in this kind intensive text-messaging study? Why, or why not, would they consider participating in a study like this one?
2. Specific to the intervention, how do you think your patients will feel about receiving text messages every day to remind them to take their medication?
   1. How can we make it as enjoyable as possible to receive daily texts?
   2. Do you think patients’ partners/family would be ok with our participants receiving daily texts for the study?

**Text Message Content**

Now we would like to shift gears a little to open up a discussion of what the text messages should say. We would really want this text messaging system to serve as the main support for people to be adherent to their anti-HIV medications. We have other things like counseling to help, but we really would like to understand how to tailor the messages to help keep people on track.

1. What kind of messages do you think would help encourage people to take their medications?
2. Do you think that patients would be interested in specific types of messages regarding taking their medications? (probes: positive/upbeat, short/ long, health-focused, overcoming barriers, drawing on support, etc)…or more interested in general reminders?
3. Are there any types of messages you think that patients would not like to receive?
4. Over the next minute or two, would you mind writing down a message or two that you think would be useful for people to remember to take their anti-HIV medications?

**Conclusions**

1. Does anyone have anything else they would like to add to our discussion group today?

2. Are there any other things that you think would be helpful for ART adherence?

Thank you very much for your time and feedback today.
